# A study of the influence of genetic variance and sex on the density and thickness of the calvarial bone in collaborative cross mice

**DOI:** 10.1002/ame2.12319

**Published:** 2023-07-13

**Authors:** Uriel Kaspersky, Roei Levy, Aysar Nashef, Fuad A. Iraqi, Yankel Gabet

**Affiliations:** ^1^ Department of Anatomy and Anthropology Tel Aviv University Tel Aviv Israel; ^2^ Department of Clinical Microbiology and Immunology, Sackler Faculty of Medicine Tel Aviv University Tel Aviv 69978 Israel; ^3^ Department of Oral and Maxillofacial Surgery Baruch Padeh medical center Poriya Israel

**Keywords:** calvarial porosity (%PoV), calvarial thickness (Ca.Th), collaborative cross mouse population, diploe, heritability, sex effect

## Abstract

**Background:**

Bone microarchitecture is affected by multiple genes, each having a small effect on the external appearance. It is thus challenging to characterize the genes and their specific effect on bone thickness and porosity. The purpose of this study was to assess the heritability and the genetic variation effect, as well as the sex effect on the calvarial bone thickness (Ca.Th) and calvarial porosity (%PoV) using the Collaborative Cross (CC) mouse population.

**Methods:**

In the study we examined the parietal bones of 56 mice from 9 lines of CC mice. Morphometric parameters were evaluated using microcomputed tomography (μCT) and included Ca.Th and %PoV. We then evaluated heritability, genetic versus environmental variance and the sex effect for these parameters.

**Results:**

Our morphometric analysis showed that Ca.Th and %PoV are both significantly different among the CC lines with a broad sense heritability of 0.78 and 0.90, respectively. The sex effect within the lines was significant in line IL111 and showed higher values of Ca.Th and %PoV in females compared to males. In line IL19 there was a borderline sex effect in Ca.Th in which males showed higher values than females.

**Conclusions:**

These results stress the complexity of sex and genotype interactions controlling Ca.Th and %PoV, as the skeletal sexual dimorphism was dependent on the genetic background. This study also shows that the CC population is a powerful tool for establishing the genetic effect on these traits.

## Graphical abstract

Using the collaborative cross mice panel we demonstrated that the porosity and thickness of the skull bone is determined by genetics and sex. (1) Selection of 9 genetically distict CC lines of inbred male and female mice, within each line all the mice are genetically identical. (2) μCT scanning and (3) 3D analysis of calvarial traits. (4) Statistical analysis of genetic (lines) and sex effect which revealed high heritability and gene‐by‐sex regulation of calvarial thickness and porosity.

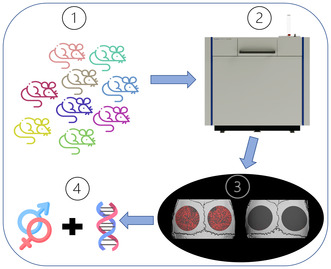

## 1 INTRODUCTION

The main purpose of the skull is to protect the soft and vulnerable brain. However, a concussion can occur when a direct impact on the skull is strong enough to cause localized flexion of the bones of the skullcap and apply focal pressure to the underlying brain tissue.^1^ Significant thickening of the calvaria has been reported in infants diagnosed with Shaken Baby Syndrome, probably as an adaptive response to strengthen the skull and prevent further damage. In addition, many medical conditions including Paget disease, fibrous dysplasia, thalassemia, and hyperostosis frontalis interna, can lead to a thickened calvaria.^2^

Reports about the possible effects of sex and genetics on thickness in human skulls are inconclusive. A study of 281 skulls from African American and Caucasian men and women reported that both race and sex have a partial effect on specific regions of the parietal bone.^3^ Another study demonstrated significant differences between male and female parietal bones, with men displaying a superior skull thickness in specific regions but inferior thickness in others.^4^ The notion of a genetic and sexual effect on parietal thickness was confirmed in another study that comprised a large number of African Americans and Caucasian Americans.^2^, ^4^, ^5^ In contrast, other studies reported that the parietal bone does not display a sexual dimorphism.^6^, ^7^, ^8^

To date, most clinical and experimental studies of the effect of genes on bone density, porosity and microarchitecture, as well as genetic determinants of osteoporosis, have focused on morphometric traits in the long bones (e.g. femur, tibia, and radius) and spinal vertebrae,^9^, ^10^, ^11^, ^12^ with far fewer studies addressing the skull bone traits.^13^, ^14^, ^15^

Because large human datasets of genetic and calvarial microarchitectural data are lacking, we conducted a microcomputed tomography analysis in the genetically diverse, inbred, and fixed Collaborative Cross population of mice. Laboratory mice are commonly used in skeletal biology, mainly because 99% of the genes in mice have analogues in humans.^16^ The Collaborative Cross (CC) mice are a population of recombinant inbred lines derived from a genetically diverse set of 8 founder strains that represent the genetic diversity of all mice. This population has been widely used for high‐resolution analysis of complex traits, with special emphasis on traits relevant to human health. Each CC line can produce large numbers of genetically identical mice, which can be phenotyped indefinitely with the high reproducibility that is especially important when investigating traits with low heritability or the effects of multiple environments.^17^

To study the genetic and sex effect on the skull, we analyzed the calvaria of mice, focusing on the parietal bone, a purely intramembranous flat bone that, in humans, forms the sides and roof of the cranium.

## 2 METHODS

### 2.1 Animals

Mice were 9‐ to 11‐week‐old young adults (male *n* = 27; female *n* = 28), from 9 different CC lines, with at least 3 males and 3 females per line (Table S1). The mice were bred and maintained at the animal facility of the Sackler Faculty of Medicine, Tel Aviv University (TAU), Israel.

### 2.2 μCT evaluation

The calvarial parietal bones (right and left) from each mouse were examined by μCT (μCT50; Scanco Medical AG). Scans were performed at a 10 μm nominal resolution in all three spatial dimensions, at 70 kV energy, 114 mA intensity and 1100 ms integration time. The signal of our Xray tube is routinely checked every 1–2 weeks using the calibration phantom provided by the manufacturer and recalibration is performed when the signal fluctuates by more than ±2.5%. The mineralized tissues were differentially segmented using a global thresholding procedure. All morphometric parameters were determined using a direct 3D approach. The morphometric parameters included calvarial thickness (Ca.Th; mm) and calvarial porosity (calculated as Ca.%PoV, and presented here as %PoV). The average of the results from the right and left parietal bones was calculated for each animal. To define the region of interest (ROI) for the measurements, we drew a cylinder of 3.8 mm in diameter at the center of each parietal bone. The cylinders defined the radial limits of the ROI, while the outer and inner periosteum and dura matter limited the outer and inner borders of the ROI. We used three‐dimensional reconstruction to check for accurate positioning of each cylinder. If ROIs were too close to the sutures or if bone fragments were included in the ROI, the position of the ROI was corrected accordingly. Importantly, in most mice the skull differs from the typical diploic structure observed in humans, and using micro‐computed tomography does not discriminate between bone marrow spaces and enlarged osteocytic lacunas (>10 μm) or perforating blood vessels. We therefore used the more general term ‘porosity’ to include all the non‐mineralized spaces within the calvarial bone.

### 2.3 Statistical analyses

Duncan's least significance range (LSR) test is presented according to our previous publications .^18^, ^19^ Data analysis was carried out using SPSS version 27. We used one‐way ANOVA to determine whether the genetic background of each CC line influenced the phenotypes and whether there was a sex effect within the lines. Broad sense heritability (*H* ^2^) and coefficient of genetic variation (CVg) were also subjected to one‐way ANOVA analysis.

### 2.4 Broad sense heritability and the genetic coefficient of variation

All phenotypes measured in this study were categorized as complex traits, which often exhibit considerable phenotypic variance (Vp) between individuals in a population. When analyzed, the phenotypic variance can be broken down into a genetic variance (Vg) and an environmental variance (Ve). Hence Vp = Vg + Ve. Heritability (*H* ^2^) can be described as the ratio between genetic variance and the phenotypic variance among individuals, i.e. *H* ^2^ = Vg/Vp, or *H* ^2^ = Vg/(Vg + Ve). The Ve can then be calculated as a measure of the variation due to environment.

The genetic Coefficient of Variation (CVg) is the ratio of the genetic standard deviation (Vg^50^) to the mean of all the CC lines. Hence, CVg = Vg^50^/Mean. CVg provides a point of reference to evaluate whether Vg values for a specific trait in the CC lines are large or small compared to the variation typically found in individual populations.^20^, ^21^

## 3 RESULTS

In this study, we used a sample of 55 male and female mice from nine genetically distinct CC lines to analyze the effect of the genotype and sex, as well as their interaction, on two phenotypic traits: Ca.Th and %Po.V.

### 3.1 Line effect

**FIGURE 1:**
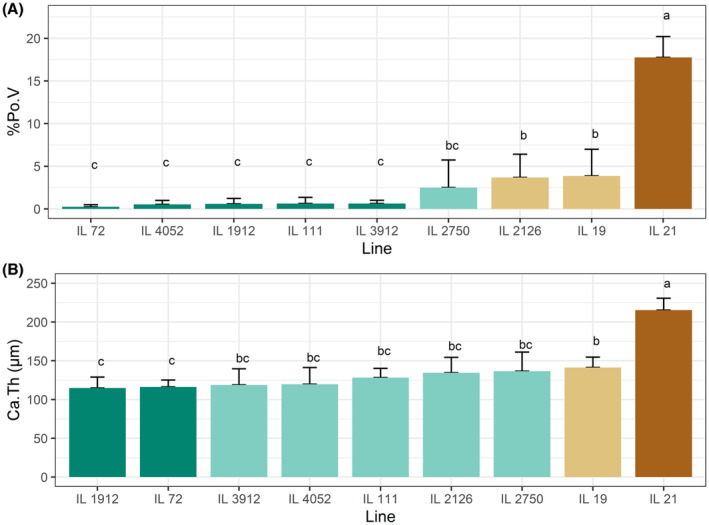
Collaborative Cross (CC) lines arranged in increasing order of the trait's mean. A, calvarial porosity (%Pov). B, calvarial bone thickness (Ca.Th). The x‐axis represents the line number; the y‐axis represents the trait means. the 3 letters above the bars represent 3 subset of lines, each group being significantly different from the other, as calculated by Duncan's least significance range test for post hoc analysis (see Table 3).

**FIGURE 2:**
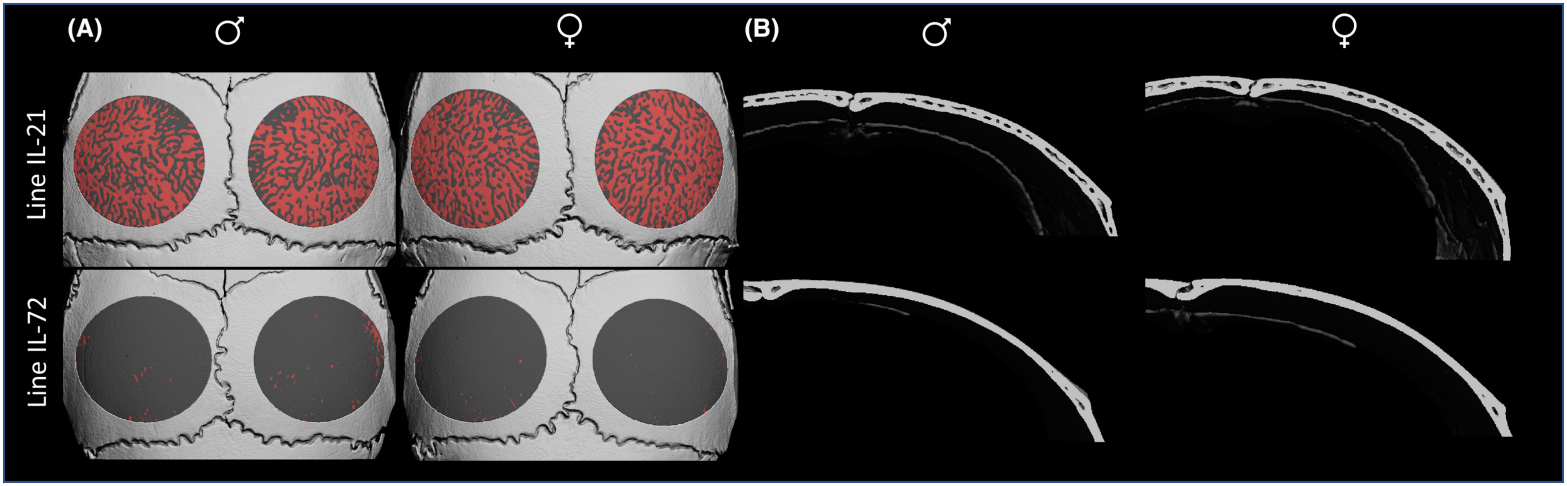
Representative μCT images of the calvaria in males and females from lines 21 and 72 showing traits differences. A, ROI is shown as transperent gray; bone marrow spaces (porosity) are shown in red. B, Frontal section of the right paraital bone depicting the differences in porosity and calvarial plate thickness.

The morphometric data on Ca.Th and %PoV indicated that the CC line had a highly significant effect, with *p* < 0.0001 for both traits (Table 1). Using Duncan's least significance range (LSR) test for post hoc analysis, we observed three groups of lines, with each group being significantly different from the others (Table 2, Figure 1). Line 21 had significantly higher values for Ca.Th and %PoV compared to all other lines. For %PoV, but not for Ca.Th, there was also a clear demarcation between the two other groups (Table 2, Figures 1 and 2). In general, there was a similar ranking for both traits across the lines. We therefore calculated the correlation between Ca.Th and %PoV and found a positive correlation of *r* = 0.87, suggesting that the thicker the calvaria, the more porous it is (Figure 2). Representative 3D images also revealed that in the lines with very thin calvarial thickness, we could not distinguish a typical diploe, i.e. trabecular bone and bone marrow spaces encompassed between two plates of cortical bone. Instead, the parietal bone appears to have one compact cortical layer with sporadic non‐mineralized spaces that are either bone marrow spaces, enlarged osteocytic lacunas or small blood vessels. As calvarial thickness gets larger, as in Line 21, a typical diploe becomes more evident (Figure 2).

**TABLE 1:** One‐way ANOVA for calvarial porosity (%PoV) and calvarial bone thickness (Ca.Th).

	Sum of squares	df	Mean square	*F*	Sig. (*p* value)
Ca.Th					
Between groups	0.047	8	0.006	19.5	<0.0001
Within groups	0.014	46	<0.0001		
Total	0.061	54			
%Po.V					
Between groups	1494	8	186.8	49.1	<0.0001
Within groups	175	46	3.8		
Total	1669	54			

Abbreviations: %PoV, calvarial porosity; Ca.Th, calvarial bone thickness; df, degrees of freedom.

**TABLE 2:** Duncan's least significance range test for post hoc analysis for calvarial porosity (%PoV) and bone thickness (Ca.Th) revealed three significantly distinct subsets for α = 0.05.

Line	*N*	%Po.V			Ca.Th (mm)		
		Subset 1	Subset 2	Subset 3	Subset 1	Subset 2	Subset 3
72	6	0.2			0.114		
4052	6	0.5			0.116		
1912	6	0.6			0.118	0.118	
3912	6	0.6			0.119	0.119	
111	7	0.6			0.128	0.128	
2750	6	2.4	2.4		0.134	0.134	
2126	6		3.6		0.136	0.136	
19	6		3.8			0.141	
21	6			17.7			0.215
Sig.		0.085	0.260	1.000	0.063	0.051	1.000

*Note*: Means for groups in homogeneous subsets are displayed. Because sample sizes are unequal, the harmonic mean of the group sizes (= 6.097) is used.

Abbreviation: Sig., statistical significance (*p* value).

### 3.2 Heritability and genetic coefficient of variation

In order to substantiate the previous observation, we calculated the heritability of Ca.Th and %PoV in our sample of 9 CC lines as 0.78 and 0.90, respectively (Table 3). The CVg values were 0.24 for Ca.Th and 1.79 for %PoV. It should be noted that because the mean was small in comparison to the genetic variance (Vg) in %PoV, the calculated value was >1 and is thus meaningless. The Vg values for %PoV and Ca.Th were 35.05 and 0.0011, respectively. The environmental variances for each parameter were 3.89 and 0.0003 for %PoV and Ca.Th, respectively (Table 3). Notably, the variations caused by genetic factors within the CC lines for %PoV and Ca.Th were 9 and 3.5 times greater than variations caused by environmental factors, respectively.

**TABLE 3:** Heritability (H^2^) and coefficient of genetic variation (CVg) calculations for calvarial porosity (%PoV) and calvarial bone thickness (Ca.Th).

Parameter	Ca.Th (mm)	%PoV
*H* ^2^	0.78	0.90
Vg	0.0011	35.051
Ve	0.0003	3.89
CVg	0.24	1.794
Trait mean	0.13602	3.3

*Note*: The average number of lines is 5.22.

Abbreviations: %PoV, calvarial porosity; Ca.Th, calvarial bone thickness; CVg, coefficient of genetic variation; *H* ^2^, estimate of broad sense heritability; Ve, environmental variance; Vg, genetic variance.

### 3.3 Sex effects within the CC lines

**FIGURE 3:**
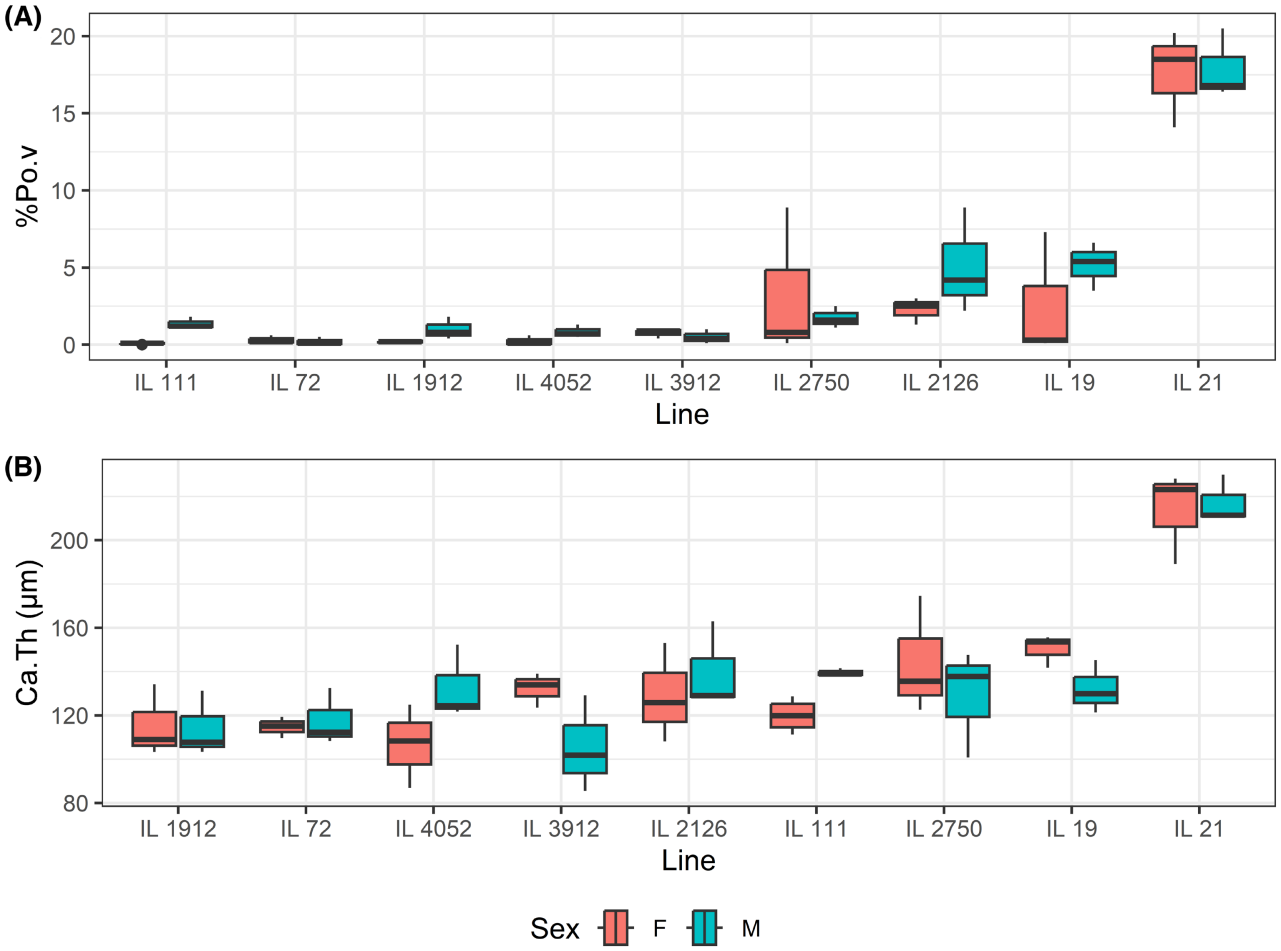
Sex effects within the CC lines. (A) Sex effect on calvarial porosity (%PoV). (B) Sex effect on calvarial bone thickness (Ca.Th). The *x*‐axis represents the lines; the *y*‐axis represents the trait means for males and females.

The cortical and trabecular bone parameters in the long bones and vertebrae frequently exhibit sexual dimorphism.^22^, ^23^ In contrast, our results revealed that in most lines, the sex effect was insignificant in the calvaria (Figure 3). One exception was that female mice in line IL111 displayed significantly higher values for Ca.Th and %PoV than their counterpart male animals (*p* = 0.009 and *p* = 0.001, respectively). Mice in line IL19 displayed a borderline sex effect (*p* = 0.091) for Ca.Th but in this case, the values in males were higher than in females (Figure 3).

## 4 DISCUSSION

The effects of heritability and sex on bone microarchitecture in long bones and vertebrae are well established, but here we demonstrate that there is also a genetic effect on the structure of the skull. Indeed, there was a significant line effect of Ca.Th and %PoV across our entire sample, and we could identify three groups of phenotypically different lines by Duncan's least significance range (LSR) test. In addition, our analyses revealed significant differences between males and females in one CC line. These findings now prompt for conducting a genome‐wide association study on a larger sample of CC mice of both sexes to identify sex‐dependent and sex‐independent putative genes that regulate calvarial bone microstructure.

As already mentioned, the main purpose of the calvaria is to protect the soft and vulnerable brain. Previous clinical studies have supported the notion of genetic regulation of parietal bone thickness, and controversial reports were published on the possible sex effect for this trait.^3^, ^4^, ^5^, ^6^, ^7^, ^8^ Notably, one study conducted in 500 Americans reported that the effect of sex was dependent on race.^5^ In this population, there was a significant sex‐related difference in parietal bone thickness for the Caucasian, but not the African Americans. Similarly, our mouse data revealed a significant sex effect in one CC line, but no differences in the rest of the lines, and with a trend for an opposite effect in one of them. This would agree with the suggestions that the effect of sex on the thickness and porosity of the calvaria is dependent on the genetic background. It is therefore reasonable to assume that the controversial data on the sexual dimorphism in human skull stems from the use of populations with different ethnicities.

The notion of gene‐by‐sex interaction is not novel. In bone biology, sex differences in the shape and dimensions of bones in humans and rodents are well documented,^22^, ^24^, ^25^ although the literature results are inconclusive. A large genome‐wide association study (GWAS) in men and women found no genome‐wide significant evidence for gene‐by‐sex interaction for bone mineral density (BMD) variation.^26^ On the other hand, other studies have claimed to demonstrate a significant interaction between sex and specific loci associated with BMD in humans.^27^, ^28^, ^29^ In laboratory animals, there are many examples of genes that have a sex‐specific skeletal role.^30^, ^31^, ^32^, ^33^ In the past, we described the sex‐specific skeletal roles of Lef1^31^ and Krox20 in mice.^30^, ^34^ In the latter case, the sexual dimorphism was associated with epigenetic changes that sex‐specifically suppressed the role of genes involved in skeletal homeostasis.^30^ Other genes such as *Sptbn1* ^35^ may even play opposite skeletal effects; while *Sptbn1* male mutants display a significantly higher whole‐body BMD, female mutants have a significantly lower BMD.^36^ In CC mice, we recently reported a gene‐by‐sex interaction for the trabecular bone in the femurs. In five lines out of 15 the bone density was higher in males, nine lines did not show any sex effect and one line showed a trend for a 2‐fold higher bone density in females.^19^ Such a sexual dimorphism was also reported in the context of the genetic regulation of calvarial bone, as evidenced by the sex‐specific phenotype (increased cranial BMD) of *Annexin1* (*AnxA1*) knockout male mice.^37^

## 5 CONCLUSION

This study demonstrates the value of the genetically diverse CC mouse population^38^, ^39^ as a powerful tool for studying the genetics of calvarial traits in a relatively small number of CC lines. We managed to show in mice that the gene and sex effects on Ca.Th are similar to those generated by large cohorts of humans. Our high‐resolution μCT analysis therefore introduces calvarial porosity as an important factor that may also contribute to the genetic and sex differences in skull resistance to fracture. Our results are in line with the hypothesis that Ca.Th and %PoV are complex traits that are controlled by multiple genetic factors as well as sex. The results of this study indicate the need for further research that uses the CC mice to define the loci and specific genes responsible for these traits.

## AUTHOR CONTRIBUTIONS

Conceptualization, Fuad A. Iraqi and Yankel Gabet; methodology, Fuad A. Iraqi, Aysar Nashef, Yankel Gabet; software, Yankel Gabet; validation, Fuad A. Iraqi, Aysar Nashef, Yankel Gabet; formal analysis, Uriel Kaspersky, Aysar Nashef, Yankel Gabet; investigation, Uriel Kaspersky, Roei Levy; resources, Fuad A. Iraqi, Yankel Gabet.; data curation, Uriel Kaspersky; writing—original draft preparation, Uriel Kaspersky, Yankel Gabet; writing—review and editing, Uriel Kaspersky, Fuad A. Iraqi, Aysar Nashef, Yankel Gabet; visualization, Uriel Kaspersky; supervision, Fuad A. Iraqi, Yankel Gabet; project administration, Yankel Gabet; funding acquisition, Yankel Gabet. All authors have read and agreed to the published version of the manuscript.

## FUNDING INFORMATION

This research was funded by Tel Aviv University starter funds and by Israel Science Foundation (ISF) grants 1822/12, 1086/17 and 1906/21 to YG, and by core funding by Tel‐Aviv University to FI and Wellcome Trust 101035/Z/13/Z.

## CONFLICT OF INTEREST STATEMENT

The authors declare no conflict of interest. Fuad A. Iraqi is an Editorial Board member of AMEM and a co‐author of this article. To minimize bias, he was excluded from all editorial decision‐making related to the acceptance of this article for publication.

## INSTITUTIONAL REVIEW BOARD STATEMENT

The animal study protocol was approved by the Institutional Ethics Committee of Tel Aviv University (protocol code MD‐13‐014, approved in 2013).

## ETHICS STATEMENT

All experimental procedures were approved by the TAU‘s Institutional Animal Care and Use Committee (IACUC M‐13‐014) in accordance with the NIH/US Animal Care and Use Protocols.

## Supporting information


Table S1

## Data Availability

The datasets supporting the results of this article will be made available upon request. The genotype of all the CC lines can be found at: http://mtweb.cs.ucl.ac.uk/mus/www/preCC/R.CD.
