# Supplementary material for: A study of the influence of genetic variance and sex on the density and thickness of the calvarial bone in collaborative cross mice
Source: Animal Model Exp Med. 2023 Jul 13;6(4):355–61. doi: 10.1002/ame2.12319 (PMC10486330; doi:10.1002/ame2.12319)
Supplement: Supplementary file 1 — Table S1 [file AME2-6-355-s001.docx]

| \| Table S1. µCT measurements for calvarial porosity (%PoV) and calvarial bone thickness (Ca.Th), and age of the mice at the time of sacrifice (weeks) for all the specimens included in this study. \| \| \| \| \| \| --- \| --- \| --- \| --- \| --- \| \| Line \| **Sex** \| **%Po.V** \| **Ca.Th (μm)** \| **Age (weeks)** \| \| 111 \| Female \| 0.13 \| 111.25 \| 11 \| \| Female \| 0.11 \| 128.7 \| 11 \| \| Female \| 0.125 \| 124.15 \| 11 \| \| Female \| 0.04 \| 115.6 \| 11 \| \| Male \| 1.2 \| 138 \| 11 \| \| Male \| 1.81 \| 138.9 \| 11 \| \| Male \| 1.12 \| 141.5 \| 11 \| \| 19 \| Female \| 0.25 \| 153.6 \| 11 \| \| Female \| 0.12 \| 155.55 \| 11 \| \| Female \| 7.27 \| 141.75 \| 11 \| \| Male \| 5.375 \| 129.9 \| 11 \| \| Male \| 3.465 \| 121.4 \| 11 \| \| Male \| 6.575 \| 145.2 \| 11 \| \| 1912 \| Female \| 0.2 \| 109 \| 10 \| \| Female \| 0.1 \| 103.3 \| 10 \| \| Female \| 0.345 \| 134.15 \| 10 \| \| Male \| 0.375 \| 103.4 \| 10 \| \| Male \| 1.795 \| 131.25 \| 10 \| \| Male \| 0.795 \| 107.8 \| 10 \| \| 21 \| Female \| 18.495 \| 228.1 \| 11 \| \| Female \| 14.12 \| 189.15 \| 11 \| \| Female \| 20.16 \| 223.15 \| 11 \| \| Male \| 16.805 \| 210.6 \| 11 \| \| Male \| 16.35 \| 211.4 \| 11 \| \| Male \| 20.52 \| 229.9 \| 11 \| \| 2126 \| Female \| 2.965 \| 153.05 \| 11 \| \| Female \| 1.335 \| 125.85 \| 10 \| \| Female \| 2.51 \| 108.15 \| 10 \| \| Male \| 8.88 \| 127.95 \| 10 \| \| Male \| 4.205 \| 129.05 \| 10 \| \| Male \| 2.24 \| 162.9 \| 10 \| \| 2750 \| Female \| 0.76 \| 135.65 \| 10 \| \| Female \| 0.1 \| 122.6 \| 11 \| \| Female \| 8.915 \| 174.5 \| 11 \| \| Male \| 1.57 \| 147.6 \| 10 \| \| Male \| 1.115 \| 100.8 \| 11 \| \| Male \| 2.465 \| 137.75 \| 11 \| \| 3912 \| Female \| 0.405 \| 123.5 \| 11 \| \| Female \| 0.985 \| 134 \| 11 \| \| Female \| 0.9 \| 138.95 \| 11 \| \| Male \| 0.14 \| 85.5 \| 11 \| \| Male \| 0.985 \| 129.15 \| 11 \| \| Male \| 0.415 \| 101.8 \| 11 \| \| 4052 \| Female \| 0.13 \| 108.35 \| 10 \| \| Female \| 0.01 \| 86.9 \| 10 \| \| Female \| 0.645 \| 124.85 \| 10 \| \| Male \| 0.67 \| 152.3 \| 11 \| \| Male \| 0.505 \| 121.75 \| 11 \| \| Male \| 1.265 \| 124.35 \| 10 \| \| 72 \| Female \| 0.125 \| 115.1 \| 9 \| \| Female \| 0.22 \| 109.6 \| 9 \| \| Female \| 0.61 \| 119.3 \| 9 \| \| Male \| 0.475 \| 132.5 \| 9 \| \| Male \| 0.075 \| 112.3 \| 9 \| \| Male \| 0.02 \| 108.35 \| 9 \| |
| --- | --- | --- | --- | --- | --- | --- | --- | --- | --- | --- | --- | --- | --- | --- | --- | --- | --- | --- | --- | --- | --- | --- | --- | --- | --- | --- | --- | --- | --- | --- | --- | --- | --- | --- | --- | --- | --- | --- | --- | --- | --- | --- | --- | --- | --- | --- | --- | --- | --- | --- | --- | --- | --- | --- | --- | --- | --- | --- | --- | --- | --- | --- | --- | --- | --- | --- | --- | --- | --- | --- | --- | --- | --- | --- | --- | --- | --- | --- | --- | --- | --- | --- | --- | --- | --- | --- | --- | --- | --- | --- | --- | --- | --- | --- | --- | --- | --- | --- | --- | --- | --- | --- | --- | --- | --- | --- | --- | --- | --- | --- | --- | --- | --- | --- | --- | --- | --- | --- | --- | --- | --- | --- | --- | --- | --- | --- | --- | --- | --- | --- | --- | --- | --- | --- | --- | --- | --- | --- | --- | --- | --- | --- | --- | --- | --- | --- | --- | --- | --- | --- | --- | --- | --- | --- | --- | --- | --- | --- | --- | --- | --- | --- | --- | --- | --- | --- | --- | --- | --- | --- | --- | --- | --- | --- | --- | --- | --- | --- | --- | --- | --- | --- | --- | --- | --- | --- | --- | --- | --- | --- | --- | --- | --- | --- | --- | --- | --- | --- | --- | --- | --- | --- | --- | --- | --- | --- | --- | --- | --- | --- | --- | --- | --- | --- | --- | --- | --- | --- | --- | --- | --- | --- | --- | --- | --- | --- | --- | --- | --- | --- | --- | --- | --- | --- | --- | --- | --- | --- | --- |
